# Prevalence and associated factors of birth defects among newborns in sub-Saharan African countries: a systematic review and meta-analysis

**DOI:** 10.11604/pamj.2020.36.19.19411

**Published:** 2020-05-14

**Authors:** Fentahun Adane, Mekbeb Afework, Girma Seyoum, Alemu Gebrie

**Affiliations:** 1 Department of Anatomy, College of Health Sciences, Addis Ababa University, Addis Ababa, Ethiopia; 2 Department of Biomedical Science, School of Medicine, Debre Markos University, Debre Markos, Ethiopia

**Keywords:** Associated factors, birth defects, prevalence, sub-Saharan African countries

## Abstract

**Introduction:**

Birth defects are the most serious causes of infant mortality and disability in sub-Saharan African countries with variable magnitude. Hence, this study was aimed to determine the pooled prevalence of birth defects and its associated risk factors among newborn infants in sub-Saharan African countries.

**Methods:**

A total of 43 eligible studies were identified through literature search from Medline (PubMed), EMBASE, HINARI, Google scholar, Science Direct, Cochrane Library and other sources. Extracted data were analyzed using STATA 15.0 statistical software. A random effect meta-analysis model was used.

**Results:**

Twenty-five studies in 9 countries showed that the pooled prevalence of birth defects was 20.40 per 1,000 births (95% CI: 17.04, 23.77). In the sub-group analysis, the highest prevalence was observed in southern Africa region with a prevalence of 43 per 1000 (95% CI: 14.89, 71.10). The most prevalent types of birth defects were musculo-skeletal system defects with a pooled prevalence of 3.90 per 1000 (95% CI: 3.11, 4.70) while the least was Down syndrome 0.62 per 1000 (95% CI: 0.40, 0.84). Lack of folic acid supplementation (95% CI: 1.95, 7.88), presence of chronic disease (95% CI: 2.00, 6.07) and intake of drugs (95% CI: 3.88, 14.66) during pregnancy were significantly associated with the birth defects.

**Conclusion:**

The prevalence of birth defects is relatively high with high degree of regional variabilities. The most common types of birth defects were musculoskeletal defects. Lack of folic acid supplementation, presence of chronic disease and intake of drugs during pregnancy were significantly associated with birth defects.

## Introduction

Birth defects (BD) are defined as a series of abnormalities of infants that occur during the pregnancy period. They are described by different terms like congenital disorders, congenital anomalies, congenital malformations and congenital abnormalities [1]. These disorders are classified as structural or functional and can result in critically damaging effects on the lives and health of infants. According to the 2015 report of World Health Organization (WHO), worldwide, congenital anomalies were identified to be the causes of death in about 276,000 newborns under one month of age every year [2]. In 2016, the figure increased to 303,000 neonates [3]. For most of the birth defects, no exact cause(s) have yet been known [3]. However, environmental teratogens, genetic factors and multifactorial inheritance are thought to be the cause of some congenital anomalies [4, 5]. Therefore, investigating these causes and risk factors may help to prevent the anomalies. At present, vaccination, dietary intake of folate or iodine, and preconception health care are available options for prevention [6, 7]. Worldwide, lifelong disability and mortality of children are the outcome of the adverse effects of birth defects. Approximately 3.3 million children under the age of five die per year, because of birth defects. Furthermore, 303,000 infants die within a month of being born because of birth defects, and 3.2 million live-born children are disabled for life, which have direct effect on children, family, health care systems and communities [8]. Although BDs are the most serious cause of infant mortality and disability in both developed and developing countries, around 94% of BDs, 95% of fatalities and 15-30% of hospital admissions of infants and children because of BDs are in low and middle income countries [9].

Worldwide, the prevalence of birth defect varies from region to region. In the United States, it has been expected that birth defects occur in 2.76% of newborns [10]. According to the European observation of Congenital Anomalies (EUROCAT), the general rate of birth defects in Europe was estimated to be 24.86 per 1,000 births during 2010-2014 [11]. In addition, according to the WHO [12] report on population-based registry in Europe the rate of multiple congenital anomalies was 51 per 1,000 live births every year. Based on the WHO report in 2013, the rates of entire structural and functional birth defects in the regions of Eastern Mediterranean and South-East Asia were 69 per 1,000 live births and 51 per 1,000 live births every year, respectively [12]. The prevalence of birth defects among newborn infants were also varied widely in sub-Saharan African countries. It was found to be 1.43 per 1000 in Gabon [13] and 68.4 per 1000 in South Africa [14]. However, there are insufficient reports on the prevalence and associated risk factors of congenital anomalies in sub-Saharan African countries, mainly due to paucity of data from the National Birth Defect Registry. The overall prevalence and associated risk factors of birth defects among newborn in sub-Saharan Africa have not yet been investigated. Therefore, the main aim of this systematic review and meta-analysis was to estimate the pooled prevalence and to identify the associated risk factors of birth defects in sub-Saharan African countries. The findings of this meta-analysis will help policy makers and other concerned bodies in planning and implementing strategies to prevent birth defects. The study could also be used as a base line for researchers to carry out investigations in related topics. The review question is: what is the best available evidence on the prevalence and associated risk factors of birth defects among newborn infants in sub-Saharan African countries?

## Methods

**Identification and study selection:** published articles and unpublished research reporting the prevalence and associated risk factors of birth defects in sub-Saharan African countries were considered. Relevant studies were identified through a literature search of Medline (PubMed), EMBASE, HINARI, Google scholar, Science Direct, Cochrane Library and other sources. The references of each incorporated article were also searched manually to optimize the search strategy. The searching of the articles was performed from the 9th of September, 2018 to the 15th of November, 2018, and it was limited to English. Unpublished studies were also searched from Google and Google Scholar. The key terms used for the search were “Prevalence” OR “Epidemiology” AND “Congenital Abnormality” OR “Congenital Abnormalities” OR “Congenital Malformation” OR “Congenital Anomaly” OR “Congenital Anomalies” OR “Birth Defect” OR “Congenital Disorders”, AND “Each country in the sub-Saharan African region”. All the literatures accessible until November 15, 2018 were included in the systematic review and meta-analysis. The systematic review and meta-analysis was conducted in agreement with the Preferred Reporting Items for Systematic reviews and Meta-Analyses (PRISMA) guideline [15].

**Inclusion criteria:** prevalence and associated factors of birth defects in sub-Saharan African countries

**Study area:** only articles conducted in sub-Saharan African countries.

**Study design:** all observational studies (cross-sectional, case controls and cohort) that contain original data reporting the prevalence and associated risk factors of birth defects in sub-Saharan African countries were considered.

**Language:** literatures published in the English language were incorporated.

**Population:** studies conducted among newborn infants were considered.

**Publication condition:** both published and unpublished articles which reported the prevalence of birth defects and associated risk factors among newborn infants in sub-Saharan African countries were considered.

**Exclusion criteria:** non-accessible articles because of un-published, un-retrievable from the internet or failures of reply to correspondences made by e-mail were excluded. In addition, studies, which did not report our outcome of interest, were excluded after reviewing their full texts.

**Data abstraction:** all the necessary data were retrieved using a consistent data extraction format made in Microsoft Excel. For the prevalence of birth defects, the data extraction format included first author, the country where the study was conducted, and the area of country where the study was carried out, publication year, study design, sample size, and prevalence of birth defects. For types of birth defects the prevalence of neural tube defects (NTD), oral-facial clefts defects (OFC), cardiovascular defects, urogenital defects, Down syndrome, gastrointestinal (GIT) defects and prevalence of unclassified birth defects were also included. For the associated risk factors, the data extraction format was prepared for each specific of the associated risk factor (maternal folic acid supplementation, maternal history of disease and maternal history of medication). We chose these variables because they are the most commonly reported associated risk factors in the studies included in this meta-analysis. In this systematic review and meta-analysis, the investigator considered variables as risk factors (maternal folic acid supplementation, maternal history of disease and maternal history of medication) if two or more studies have mentioned them as risk factors. For every associated risk factor, to compute the odds ratio, the data from the primary studies were extracted in the form of two by two tables.

**Outcome measurements:** this systematic review and meta-analysis have two major outcomes. The primary outcome was prevalence of birth defects among newborn infants in sub-Saharan African countries. The second outcome of the study was risk factors of birth defects in the countries. The prevalence was calculated by dividing the number of infants born with birth defects to the total number of born infants in the study period who have been included in the study (sample size) multiplied by 1000.

**Quality assessment:** to evaluate the quality of the studies included in this review, the researcher applied the Newcastle-Ottawa Scale tool as modified for cross-sectional studies´ quality assessment [16]. The tool consists of three major parts; the first part has five stars and assesses the methodological quality of each study. The second part of the tool assesses the comparability of the studies. The last part determines the quality of the original articles with respect to their statistical analysis. Using the tool as a checklist, the qualities of each of the original articles were evaluated. Articles with medium (fulfilling 50% of quality assessment criteria) and high quality (≥6 out of 10 scales) were considered to be included for the analysis.

**Statistical analysis:** the required data were collected using a Microsoft Excel format and analyzed by using STATA Version 15.0 software. The original articles were presented using tables and forest plot. The researcher calculated the standard error of prevalence for each original article by the binomial distribution formula. Heterogeneity among the reported prevalence of studies was checked by using heterogeneity χ2 test, I2 test and the p-values [17]. The above statistic tests indicated that there was a significant heterogeneity among the studies (I2= 97.5%, p < 0.001). As a result, a random effects meta-analysis model was applied to estimate the DerSimonian and Laird's pooled effect. In addition, univariate meta-regression model was conducted by taking publication year and sample size of the studies to discover the probable source of heterogeneity but none of them was statistically significant. Possible publication bias was also evaluated objectively by using Egger's correlation and Begg's regression intercept tests at 5% significant level respectively [18, 19]. The outcome of these assessments proposed that for a likely existence of a significant publication bias (p < 0.001 in Egger's test), the last effect size was determined by using Duval and Tweedie's Trim and Fill analysis in the Random-effects model. Furthermore, to reduce the random discrepancies between the point estimates of the primary study, subgroup analysis was carried out based on region of studies.

## Results

**Figure 1:**
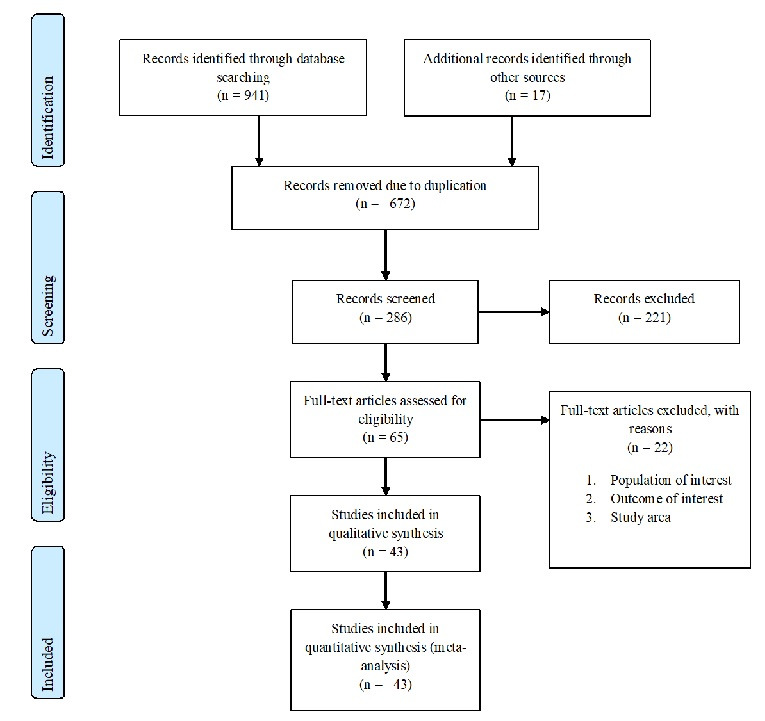
Flow chart describing the selection of studies for the systematic review and meta-analysis of prevalence and associated factors of birth defects among newborn in sub-Saharan African countries; 2018 (identified screened, eligible and included studies); articles may have been excluded for more than one reason

**Search results:** a total of 958 records regarding prevalence and associated factors of birth defects in sub-Saharan African countries were retrieved from the databases of Medline (PubMed), EMBASE, HINARI, Google scholar, Science Direct, Cochrane Library and other sources described above. From these preliminary records, 672 articles were excluded due to duplication. From the remaining 286 articles, 221 articles were excluded as they were found to be non-applicable to this review after assessing their titles and abstracts. The remaining 65 full text articles were then accessed, and assessed for eligibility based on the preset criteria, which resulted in further exclusion of 22 articles primarily due to the study population and outcome of interest. Among these, fourteen of the studies were conducted in countries other than sub-Saharan African countries: Indian, Iran, Brazil, Canada, China, Italy, Latin America, Pakistan, Spain, Saudi Arabia and Egypt. Three of the studies were conducted in: Tanzania, South Africa and Ugandan and excluded because their study population were not among newborn infants. The remaining five studies were conducted in different parts of Ethiopia and excluded because of the study population and unreported outcome of interest. Finally, 43 studies were found to be eligible and incorporated in the meta-analysis. Among the eligible studies, 25 studies were used for overall prevalence of birth defects. For prevalence of sub type of birth defects, 16 additional studies were included and 2 case control studies were also included for associated risk factors of birth defects (Figure 1).

**Characteristics of original articles:** Table 1, Table 1 (suite), Table 1 (suite 1), Table 1 (suite 2) summarizes the characteristics of the original articles included in this systematic review and meta-analysis. The study designs for all of the researches were cross sectional study design. Besides, these studies were conducted from 1961 to 2018. The quality score of the researches varied from 6 to 8 out of 10 points. Twenty-five published researches from nine sub-Saharan African countries were used for analysis of pooled prevalence of birth defects. The studies were conducted in Nigeria [20-30], Kenya [31], Tanzania [32], Democratic Republic of Congo [33], South Africa [14, 34-36], Gabon [13], Uganda [37, 38], Ghana [39] and Ethiopia [40-42]. The sample size ranged from 600 in South Africa [35] to 319776 in Ethiopia [42]. The highest prevalence was 85.41 per 1000 in Nigeria [27] while the lowest was 1.43 in Gabon [13]. Fifteen published studies from six sub-Saharan African countries were used for analysis of pooled prevalence of musculoskeletal system defects. The researches were conducted in Nigeria [22-25, 27, 29, 30], Democratic Republic of Congo [33], Tanzania [32], South Africa [6, 34], Kenya [31] and Ethiopia [40-42]. The sample size ranged from 843 in Nigeria [27] to 319 776 in Ethiopia [42]. The highest prevalence was 24.9 per 1000 in Nigeria [27] while the lowest was 0.37 per 1000 also in Nigeria [25]. The remaining types of birth defects are as described in Table 1, Table 1 (suite), Table 1 (suite 1), Table 1 (suite 2). For associated risk factors of birth defects four researches from two sub-Saharan African countries were used. The studies were conducted in Tanzania [43, 44] and in Ethiopia [40, 45] (Table 2).

**Table 1:** Descriptive summary of 25 studies reporting the prevalence of birth defects and additional studies reporting the prevalence of sub types of birth defects among newborn in sub-Saharan African countries included in the systematic review and meta-analysis

**Birth defects**	**Author**	**Publication year**	**Country**	**African Region**	**Sample size**	**Case**	**Quality score (10 pts)**	**Prevalence Per 1000(95% CI)**
	Abbey M *et al.*	2017	Nigeria	Western Africa	7,670	159	7	20.73(17.54,23.92)
	Adane and Seyoum	2018	Ethiopia	Eastern Africa	19,650	317	8	16.13(14.37,17.89)
	Ahuka *et al.*	2006	D/R of Congo	Central Africa	8,824	36	6	4.08(2.75,5.41)
	Anyanwu L-JC *et al.*	2015	Nigeria	Western Africa	1,456	41	7	28.16(19.66, 36.66)
	Delport S *et al.*	1995	South Africa	Southern Africa	17, 351	1,187	6	68.41(64.65, 72.17)
	Ekanem *et al.*	2008	Nigeria	Western Africa	127, 929	452	8	3.53(3.21, 3.850)
	Ekanem TB *et al.*	2011	Nigeria	Western Africa	39,693	125	8	3.15(2.60, 3.70)
	Ekwochi *et al.*	2017	Nigeria	Western Africa	5,830	38	7	6.52(4.45, 8.59)
	Iroha *et al.*	2001	Nigeria	Western Africa	22,288	353	6	15.84(14.20, 17.48)
	Kishimba RS *et al.*	2015	Tanzania	Eastern Africa	27, 230	77	6	2.83(2.20, 3.46)
	Kromberg J and Jenkins T	1982	South Africa	Southern Africa	29, 633	435	7	14.68(13.31, 16.05)
	Liu *et al.*	2014	South Africa and Zambia	Southern Africa	600	36	6	60.00(41.00, 79.00)
	Mekonen *et al.*	2015	Ethiopia	Eastern Africa	1,516	32	7	21.11(13.87, 28.35)
	Mombo *et al.*	2017	Gabon	Eastern Africa	3,500	5		1.43(0.18, 2.68)
	Muga R *et al.*	2009	Kenya	Eastern Africa	7,355	207	7	28.14(14.88, 41.14)
	Mukhtar-Yola M *et al.*	2005	Nigeria	Western Africa	13,619	75	6	5.51(4.27, 6.27)
	Ndibazza J *et al.*	2011	Uganda	Central Africa	2,365	180	7	76.11(65.42, 86.80)
	Nuertey *et al.*	2017	Ghana	Western Africa	37,303	161	8	4.32(3.10, 5.20)
	Obu HA *et al.*	2012	Nigeria	Western Africa	607	17	6	28.01(14.88, 41.14)
	Ochoga *et al.*	2018	Nigeria	Western Africa	843	72	8	85.41(66.54, 104.28)
	Onankpa and Adamu	2014	Nigeria	Western Africa	6,578	24	6	3.65(2.19, 5.11)
	Onyearugha C and Onyire B	2014	Nigeria	Western Africa	14,446	60	7	4.15(3.10, 5.20)
	Simpkiss M and Lowe A	1961	Uganda	Central Africa	2,068	112	6	54.16(44.41, 63.91)
	Taye *et al.*	2016	Ethiopia	Eastern Africa	319,776	6076	8	19.00(18.53, 19.47)
	Venter P *et al.*	1995	South Africa	Southern Africa	7,617	234	6	30.72(26.84, 34.60)

**Table 1 (suite):** Descriptive summary of 25 studies reporting the prevalence of birth defects and additional studies reporting the prevalence of sub types of birth defects among newborn in sub-Saharan African countries included in the systematic review and meta-analysis

Typeof birth defect	Author	Publication year	Country	African Region	Sample size	Case	Quality score (10 pts)	Prevalence Per 1000(95% CI)
**Oro-facial clefts defects**	Adane and Seyoum	2018	Ethiopia	Eastern Africa	19650	86	8	2.60(1.89, 3.31)
	Agbenorku *et al.*	2011	Ghana	Western Africa	4000	25	7	6.25(3.81, 8.69)
	Butali *et al.*	2014	Nigeria	Western Africa	4600000	2197	8	0.48(0.46, 0.50)
	Ekanem *et al.*	2008	Nigeria	Western Africa	127929	56	8	0.44(0.33, 0.55)
	Eshete *et al.*	2017	Ethiopia	Eastern Africa	18811316	8232	7	0.44(0.43, 0.45)
	Kesande *et al.*	2014	Uganda	Central Africa	25985	20	7	0.77(0.43,1.11)
	Kishimba RS *et al.*	2015	Tanzania	Eastern Africa	27230	11	6	0.40(0.16, 0.64)
	Kromberg J and Jenkins T	1982	South Africa	Southern Africa	29633	9	7	0.30(0.10, 0.50)
	Njamnshi *et al.*	2008	Cameroon	Central Africa	52710	98	7	1.86(1.49, 2.23)
	Onankpa and Adamu	2014	Nigeria	Western Africa	6578	5	6	0.76(0.09, 1.43)
	Rakotoarison *et al.*	2012	Madagascar	Eastern Africa	150973	73	8	0.48(0.37, 1.27)
	Suleiman *et al.*	2005	Sudan	Northeastern Africa	15890	13	7	0.82(0.37, 1.27)
	Taye *et al.*	2016	Ethiopia	Eastern Africa	319776	2076	8	6.49(6.21, 6.77)
**Neural tube defects**	Adane and Seyoum	2018	Ethiopia	Eastern Africa	19650	103	8	5.24(4.23, 6.25)
	Ahuka *et al.*	2006	D/R of congo	Central Africa	8824	17	6	1.93(1.01, 2.85)
	Buccimazza *et al.*	1994	South Africa	Southern Africa	516252	606		1.17(1.08, 1.26)
	Ekanem *et al.*	2008	Nigeria	Western Africa	127929	111	8	0.87(0.71, 1.03)
	Ekanem TB *et al.*	2011	Nigeria	Western Africa	39693	52	8	1.31(0.95, 1.67)
	Ekwochi *et al.*	2017	Nigeria	Western Africa	5830	14	7	2.40 (1.14,3.66)
	Gedefaw *et al.*	2018	Ethiopia	Eastern Africa	8677	109	8	12.56(10.22, 14.90)
	Iroha *et al.*	2001	Nigeria	Western Africa	22288	80	6	3.59(2.80, 4.38)
	Kishimba RS *et al.*	2015	Tanzania	Eastern Africa	27230	39	6	1.43(0.98, 1.88)
	Kromberg J and Jenkins T	1982	South Africa	Southern Africa	29633	73	7	2.46(1.90, 3.02)
	Mekonen *et al.*	2015	Ethiopia	Eastern Africa	1516	22	7	14.51(8.49, 20.53)
	Muga R *et al.*	2009	Kenya	Eastern Africa	7355	32	7	4.35(2.85, 5.85)
	Mukhtar-Yola M *et al.*	2005	Nigeria	Western Africa	13619	22	6	1.62(0.94, 2.30)
	Ochoga *et al.*	2018	Nigeria	Western Africa	843	28	8	33.21(21.11, 45.31)
	Omer *et al.*	2016	Sudan	Eastern Africa	36785	103	7	2.80(2.26, 3.34)
	Onankpa and Adamu	2014	Nigeria	Western Africa	6578	7	6	1.06(0.27, 1.85)
	Onyearugha C and Onyire B	2014	Nigeria	Western Africa	14446	8	7	0.55(0.17, 0.93)
	Sayed *et al.*	2008	South Africa	Southern Africa	79587	122	8	1.53(1.26, 1.80)
	Taye *et al.*	2016	Ethiopia	Eastern Africa	319776	1873	8	5.86(5.60, 6.12)
	Ugwu *et al.*	2007	Nigeria	Western Africa	7388	7	7	0.95(0.25, 1.65)
	Venter P *et al.*	1995	South Africa	Southern Africa	7617	33	6	4.33(2.86, 5.80)

**Table 1 (suite 1):** Descriptive summary of 25 studies reporting the prevalence of birth defects and additional studies reporting the prevalence of sub types of birth defects among newborn in sub-Saharan African countries included in the systematic review and meta-analysis

**Musculoskeletal defects**	Adane and Seyoum	2018	Ethiopia	Eastern Africa	19650	21	8	1.06(0.61, 1.51)
	Ahuka *et al.*	2006	D/R of Congo	Central Africa	8824	11	6	1.25(0.51, 1.99)
	Ekanem *et al.*	2008	Nigeria	Western Africa	127929	132	8	1.03(0.85, 1.21)
	Ekanem TB *et al.*	2011	Nigeria	Western Africa	39693	57	8	1.44(1.07, 1.81)
	Ekwochi *et al.*	2017	Nigeria	Western Africa	5830	67	7	11.50(8.76, 14.24)
	Iroha *et al.*	2001	Nigeria	Western Africa	22288	49	7	2.19(1.58, 2.80)
	Kishimba RS *et al.*	2015	Tanzania	Eastern Africa	27230	19	6	0.69(0.38, 1.00)
	Kromberg J and Jenkins T.	1982	South Africa	Southern Africa	29633	353	7	11.90(10.67, 13.13)
	Mekonen *et al.*	2015	Ethiopia	Eastern Africa	1516	4	7	2.60(0.04, 5.16)
	Muga R *et al.*	2009	Kenya	Eastern Africa	7355	127	7	17.27(14.29, 20.25)
	Mukhtar-Yola M *et al.*	2005	Nigeria	Western Africa	13619	5	6	0.37(0.05, 0.69)
	Ochoga *et al.*	2018	Nigeria	Western Africa	843	21	8	24.90(14.38, 35.42)
	Onyearugha C and Onyire B	2014	Nigeria	Western Africa	14446	19	7	1.30(0.71, 1.89)
	Taye *et al.*	2016	Ethiopia	Eastern Africa	319776	535	8	1.67(1.53, 1.81)
	Venter P *et al.*	1995	South Africa	Southern Africa	7617	142	6	18.64(15.60, 21.68)
**Urogenital defects**	Adane and Seyoum	2018	Ethiopia	Eastern Africa	19650	9	8	0.46(0.16, 0.76)
	Ahuka *et al.*	2006	D/R of Congo	Central Africa	8824	6	6	0.68(0.14, 1.22)
	Ekanem *et al.*	2008	Nigeria	Western Africa	127929	83	8	0.65(0.51, 0.79)
	Ekanem TB *et al.*	2011	Nigeria	Western Africa	39693	7	8	0.18(0.05, 0.79)
	Ekwochi *et al.*	2017	Nigeria	Western Africa	5830	10	7	1.72(0.66, 2,78)
	Iroha *et al.*	2001	Nigeria	Western Africa	22288	34	6	1.53(1.02, 2.04)
	Mekonen *et al.*	2015	Ethiopia	Eastern Africa	1516	5	7	3.30(0.41, 6.19)
	Mukhtar-Yola M *et al.*	2005	Nigeria	Western Africa	13619	11	6	0.81(0.33, 1.29)
	Ochoga *et al.*	2018	Nigeria	Western Africa	843	5	8	5.93(0.75, 11.11)
	Taye *et al.*	2016	Ethiopia	Eastern Africa	319776	123	8	0.38(0.31, 0.45)
	Venter P *et al.*	1995	South Africa	Southern Africa	7617	10	6	1.31(0.50, 2.12)

**Table 1 (suite 2):** Descriptive summary of 25 studies reporting the prevalence of birth defects and additional studies reporting the prevalence of sub types of birth defects among newborn in sub-Saharan African countries included in the systematic review and meta-analysis

**Down syndrome**	Adane and Seyoum	2018	Ethiopia	Eastern Africa	19650	10	8	0.51(0.19, 0.83)
	Ekanem *et al.*	2008	Nigeria	Western Africa	127929	15	8	0.12(0.06, 0.18)
	Iroha *et al.*	2001	Nigeria	Western Africa	22288	10	6	0.45(0.17, 0.73)
	Kishimba RS *et al.*	2015	Tanzania	Eastern Africa	27230	5	6	0.18(0.02, 0.34)
	Kromberg and Zwane	1993	South Africa	Southern Africa	24000	40	7	1.67(1.15, 2.19)
	Muga R *et al.*	2009	Kenya	Eastern Africa	7355	12	7	1.63(0.71, 2.55)
	Mukhtar-Yola M *et al.*	2005	Nigeria	Western Africa	13619	11	6	0.81(0.33, 1.29)
	Ochoga *et al.*	2018	Nigeria	Western Africa	843	10	8	11.86(4.55, 19.17)
	Taye *et al.*	2016	Ethiopia	Eastern Africa	319776	124	8	0.39(0.32, 0.46)
	Venter *et al.*	1995	South Africa	Southern Africa	7617	21	6	2.76(1.58, 3.94)
**Gastrointestinal defects**	Adane and Seyoum	2018	Ethiopia	Eastern Africa	19650	9	8	0.46(0.16, 0.76)
	Ekanem TB *et al.*	2011	Nigeria	Western Africa	39693	4	8	0.10(0.00, 0.20)
	Ekwochi *et al.*	2017	Nigeria	Western Africa	5830	22	7	3.77(2.20, 5.34)
	Iroha *et al.*	2001	Nigeria	Western Africa	22288	87	6	3.90(3.08, 4.72)
	Mekonen *et al.*	2015	Ethiopia	Eastern Africa	1516	5	7	3.30(0.41, 6.19)
	Mukhtar-Yola M *et al.*	2005	Nigeria	Western Africa	13619	29	6	2.13(1.36, 2.90)
	Ochoga *et al.*	2018	Nigeria	Western Africa	843	21	8	24.91(14.39, 35.43)
	Onankpa and Adamu	2014	Nigeria	Western Africa	6578	4	6	0.61(0.01, 1.21)
	Onyearugha C and Onyire B	2014	Nigeria	Western Africa	14446	22	7	1.52(0.88, 2.16)
	Taye *et al.*	2016	Ethiopia	Eastern Africa	319776	289	8	0.90(0.80, 1.00)
	Venter P *et al.*	1995	South Africa	Southern Africa	7617	9	6	1.18(0.41, 1.95)
**Cardiovascular defects**	Abid *et al.*	2014	Tunisia	North Africa	37294	255	7	6.84(6.00, 7.68)
	Adane and Seyoum	2018	Ethiopia	Eastern Africa	19650	38	8	1.93(1.32, 2.54)
	Ekwochi *et al.*	2017	Nigeria	Western Africa	5830	9	7	1.54(0.53, 2.55)
	Iroha *et al.*	2001	Nigeria	Western Africa	22288	40	6	1.79(1.24, 2.34)
	Mukhtar-Yola M *et al.*	2005	Nigeria	Western Africa	13619	5	6	0.37(0.05, 0.69)
	Onankpa and Adamu	2014	Nigeria	Western Africa	6578	7	6	1.06(0.27, 1.85)
	Sani *et al.*	2007	Nigeria	Western Africa	1499	122	7	81.39(67.55, 95.23)
	Taye *et al.*	2016	Ethiopia	Eastern Africa	319776	626	8	1.96(1.81, 4.84)
**Unspecified defects**	Adane and Seyoum	2018	Ethiopia	Eastern Africa	19650	17	8	0.87(0.46, 1.28)
	Iroha *et al.*	2001	Nigeria	Western Africa	22288	43	6	1.93(1.35, 2.51)
	Mukhtar-Yola M *et al.*	20105	Nigeria	Western Africa	13619	6	6	0.44(0.09, 0.79)
	Taye *et al.*	2016	Ethiopia	Eastern Africa	319776	150	8	0.47(0.39, 0.56)

**Table 2:** Descriptive summary of 4 studies reporting the associated risk factors of birth defects among newborn in sub-Saharan African countries included in the systematic review and meta-analysis

Authors	Publication year	Country	Region	Variables		Birth defects	
						Yes	No
Mashuda *et al.*	2014	Tanzania	Mwanza	Maternal folic acid supplementation	Yes	8	59
					No	122	253
Adane and Seyoum	2018	Ethiopia	Northwest Ethiopia		Yes	33	155
					No	71	62
Taye *et al.*	2018	Ethiopia	Addis Ababa and Amhara Region		Yes	2	22
					No	205	185
Kishimba *et al.*	2015	>Tanzania	Dar Es Salaam		Yes	73	252
					No	25	48
Adane and Seyoum	2018	Ethiopia	Northwest Ethiopia	Maternal illness	Yes	62	105
					No	32	147
Taye *et al.*	2018	Ethiopia	Addis Ababa and Amhara Region		Yes	24	13
					No	183	194
Kishimba *et al.*	2015	Tanzania	Dar Es Salaam		Yes	14	11
					No	86	289
Adane and Seyoum	2018	Ethiopia	Northwest Ethiopia	Maternal history of medication	Yes	73	31
					No	31	132
Taye *et al.*	2018	Ethiopia	Addis Ababa and Amhara Region		Yes	35	8

### Meta-analysis

**Figure 2:**
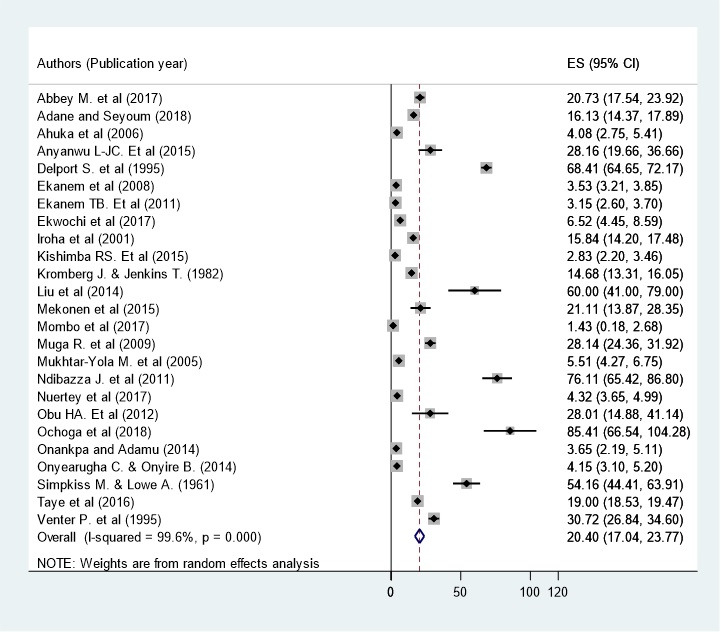
Forest plot of the sub-group showing the pooled prevalence of birth defects in sub-Saharan African countries, 2018

**Prevalence of birth defects:** twenty five studies in 9 sub-Saharan African countries showed that the prevalence of birth defects was 20.40 per 1,000 births (95% CI: 17.04-23.77) [13, 14, 20-42] (Figure 2). However, considerable heterogeneity was found across the studies as revealed by I2 statistic (I2 = 96.6, p value <0.000). Therefore, a random effect model was used to estimate the pooled prevalence of birth defects in sub-Saharan African countries. A uni-variate meta-regression model was also carried out to identify the possible sources of heterogeneity, by considering factors such as publication year and sample size although none of these variables was found to be statistically significant. However, Beggs' and Eggers' tests exposed the presence of statistically significant publication bias of p = 0.008 and p = 0.044, respectively. Thus, Trim and Fill analysis were performed to correct the final pooled estimate.

**Figure 3:**
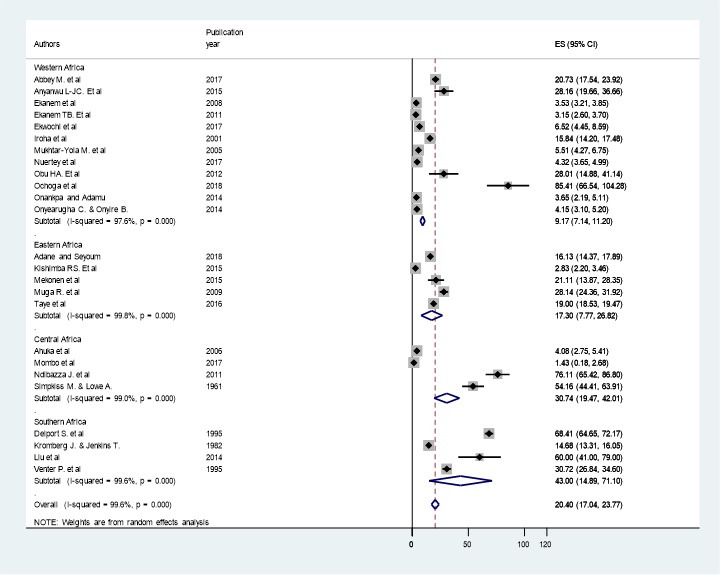
Forest plot of the sub-group analysis of prevalence of birth defects in sub-Saharan African countries, 2018

**Subgroup analysis:** in this meta-analysis, subgroup analysis was carried out based on the regions where the studies were conducted. The highest prevalence was observed in Southern Africa region with a prevalence of 43 per 1000 (95% CI: 14.89, 71.10) followed by Central African 30.74 per 1000 (95% CI: 19.47, 42.01), Eastern Africa 17.30 per 1000 (95% CI: 7.77, 26.82) and Western Africa, 9.17 per 1000 (95% CI: 7.14, 11.20) (Figure 3).

**Types of birth defects:** among the types of birth defects in sub-Saharan African countries, the most frequent types of BD were musculoskeletal systems defects with a pooled prevalence of 3.90 per 1000 (95% CI: 3.11, 4.70), followed by neural tube defects 2.98 per 1000 (95% CI: 2.25, 3.71), cardiovascular system defects (CVSDs) 2.83 per 1000 (95% CI: 1.56, 4.11), gastrointestinal defects 1.50 per 1000 (95% CI: 1.00, 2.01), oro-facial clefts (OFCs) 1.27 per 1000 (95% CI: 1.6, 1.48), unspecified birth defects 0.86 per 1000(95% CI: 0.38, 1.34), urogenital system defects 0.69 per 1000 (95% CI: 0.47, 0.91) and Down syndrome 0.62 per 1000 (95% CI: 0.40, 0.84) (Table 3).

**Table 3:** table depicting the pooled prevalence of different types of birth defects in sub-Saharan African Countries, 2018

Types of birth defects	Pooled prevalence per 1000	(95% CI :)
Musculoskeletal systems defects	3.90	(3.11, 4.70)
Neural tube defects	2.98	(2.25, 3.71)
Cardiovascular system defects	2.83	(1.56, 4.11)
Gastrointestinal defects	1.50	(1.00, 2.01)
Oro-facial clefts	1.27	(1.6, 1.48)
Unspecified birth defects	0.86	(0.38, 1.34)
Urogenital system defects	0.69	(0.47, 0.91)
Down syndrome	0.62	(0.40, 0.84)

**Figure 4:**
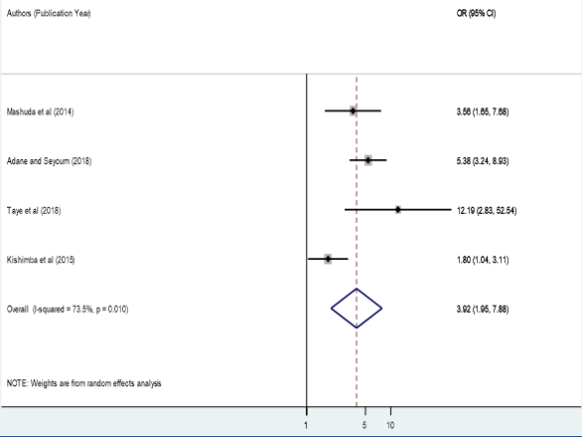
Forest plot depicting pooled odds ratio (log scale) of the associations between prevalence of birth defects and maternal folic acid supplementation, 2018

**Figure 5:**
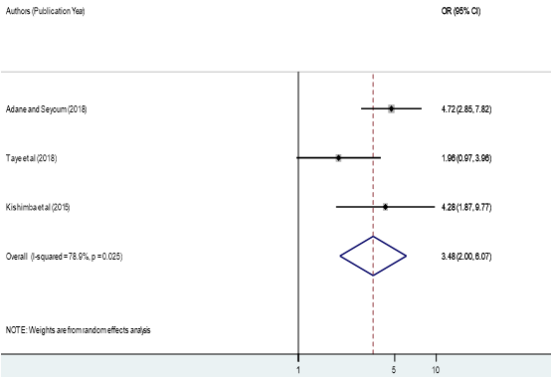
Forest plot depicting pooled odds ratio (log scale) of the associations between prevalence of birth defects and maternal illness, 2018

**Figure 6:**
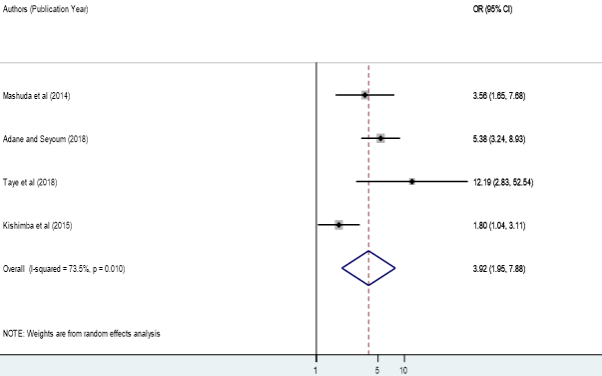
Forest plot depicting pooled odds ratio (log scale) of the associations between prevalence of birth defects and maternal history of medication, 2018

**Factors associated with birth defects:** the associations of maternal folic acid supplementation, maternal illness and maternal history of medication during pregnancy with birth defects were analyzed in four analyzable studies [40, 43-46]. Sensitivity analyses were also performed for each of the factors, but none of these was found to be significant. Therefore, from the four studies, we found that maternal folic acid supplementation during pregnancy was significantly associated with birth defects among newborn infants, odds ratio 3.92 (95% CI 1.95, 7.88) (Figure 4). In epidemiological expressions, this shows us that infants born from mothers who did not have folic acid supplementation during pregnancy were 3.92 times more likely to have birth defects as compared to those who had folic acid supplementation during pregnancy. Three studies also pointed out that the presence of maternal illness during pregnancy was significantly associated with the birth defects among born infants, odds ratio 3.48 (95% CI 2.00, 6.07) (Figure 5). This implies that infants of mothers who had a disease during pregnancy were 2.19 times more likely to have birth defects. Finally, two studies showed that maternal history of medication during pregnancy was significantly associated with birth defects among born infants, odds ratio 7.54 (95% CI 3.88, 14.66) (Figure 6). This indicated that infants of mothers who took drugs during pregnancy were 7.54 times more likely to have birth defects.’

## Discussion

This meta-analysis is the first of its kind in sub-Saharan African countries to estimate the pooled prevalence and associated risk factors of birth defects among newborn infants. The study has found that the overall prevalence of birth defects in sub-Saharan African countries among newborn infants is 20.4 per 1000 (95% CI: 17.04, 23.77%). This finding is in line with a study in the northeast region of Cairo, Egypt, which has reported a BD prevalence of 20 per 1000 [46] among newborn infants and children. However, the prevalence of BDs observed in the present study is slightly less than another study conducted in Europe which reported prevalence values of 23.9 per 1000 and 24.86 per 1000 [11, 47]. A higher prevalence of birth defects with 41 per 1000 has been described in a study performed in Pakistan [48]. Furthermore, based on the WHO report in 2013, the rates of total structural and functional birth defects were higher in the regions of Eastern Mediterranean and South-East Asia with respective prevalence rates of 69 per 1,000 and 51 per 1,000 live births every year [12]. There could be several reasons for the heterogeneity of the prevalence rates among the various studies as compared with the present study. Higher prevalence rates of BD in those studies in Europe may be due to well organized and better birth registry system compared to sub-Saharan African countries. As a result, the possibility of data loss may be greater in sub-Saharan African countries than Europe. Difference in study design, population sampling and ethnicity may also be factors that contribute for the higher prevalence rates in the above studies. On the other hand, a lower frequency of birth defects (12.5 per 1000 live births) has also been reported by a research carried out in India where the data were collected from a smaller sample size in a single hospital [49].

The subgroup analysis of this study showed that the prevalence of birth defects among newborn infants significantly varies across regions of the sub-Saharan African countries. The highest prevalence of birth defects was observed in Southern Africa region, 43 per 1000 (95%CI: 14.89, 71.1%) followed by the Central Africa region, 30.74 per 1000 (95% CI: 19.47, 42.01%) and Eastern Africa 17.3 per 1000 (95% CI: 7.77, 26.82%), while the lowest prevalence was observed in the Western Africa region with a prevalence of 9.17 per 1000 (95%CI: 7.14, 11.2%). The present finding is in trajectory to the report of the study conducted on the prevalence of common birth defects in South African infants [34]. This might be because of the reason that birth defect is a principal disorder which is much more common in the South African black populations as found by Stevenson AC [50]. This could be explained by the fact that in South Africa environmental teratogens are much more common than other sub-Saharan African countries. It is because of better economic status of South Africa than other sub-Saharan African countries. The other possible justification for this discrepancy could be due to the variation in sample size for the different studies conducted across the regions.

The most common types of birth defect were musculoskeletal systems defects followed by neural tube defects while the least frequent were urogenital system defects and down syndrome. Comparable sequence of the organ system defects have been described in a study conducted in southern Vietnam and where the most common types of BD were musculoskeletal system defects [51]. However, in a previous systematic review and meta-analysis on types of birth defects conducted in Iran [52], urogenital anomalies were the most frequent type of birth defects. This could be explained by the fact that the risk factors for the type of birth defects may vary from region to region [12]. The present study shows that there are significant associations between the prevalence of BDs and maternal history of medication during pregnancy, lack of maternal folic acid supplementation and presence of chronic disease during pregnancy. Infants born from mothers who have history of medication during pregnancy were 7.54 times more likely to have a birth defects compared to infants born from mothers who did not have history of medication drug during pregnancy. This finding is in line with that of another study which reported that maternal use of non-steroidal anti-inflammatory drugs during pregnancy is a risk factor for congenital anomalies [53]. It is also supported by an experimental study conducted to evaluate the toxicological consequences of chloroquine and ethanol on the developing rat fetus which has shown a teratogenic effect of anti-malarias causing structural birth defects such as cleft palate, wrist drop, clubbed foot and brain liquefaction [54]. It, therefore, appears that pregnant women taking both prescribed and non-prescribed drugs which have the ability to pass through placental barrier may disturb the normal organogenesis and histogenesis of the developing embryos as suggested by Nelson MM and Forfar JO [55].

Infants born from mothers who had no folic acid supplementation during pregnancy were 3.92 times more likely to have birth defects compared to those born from mothers who had folic acid supplementation. This finding is in line with a study which reported that folic acid supplementation lowers the risk of birth defects [56]. The present finding is also in agreement with a study conducted in Texas, which reported that maternal folic acid supplementation considerably reduces the magnitude of NTDs [57]. Maternal folic acid supplementation, particularly one month before conception and all over the first trimester, appreciably diminishes BDs, primarily NTDs [58-61]. This could be explained by the fact that folic acid supplementation with vitamin B12 is very important for the synthesis of nucleic acid, lipids, and proteins, necessary for cell division, differentiation and migration [62, 63]. Iron folate/folic acid is also very important in amino acids metabolisms that are required for DNA and RNA synthesis and plays an important role as an antioxidant agent. As a result, it is necessary to make folic acid accessible to all pregnant women to prevent birth defects [62].

Infants born from mothers who had a disease during pregnancy were 3.48 times more likely to have birth defects as compared to infants born from mothers who did not have diseases. The presence of maternal illness during pregnancy as a risk factor for birth defects is previously acknowledged by other study [64]. The present result is also in line to the systematic review and meta-analysis which have reported previously as maternal obesity and gestational diabetes are risk factors for birth defects [65, 66]. This may be explained by the fact that maternal chronic disease like hypertension and diabetes during pregnancy compromises utero-placental circulation, which affects the development of the embryo [67]. Hypertension during pregnancy had also been implicated to significantly increase the risk for other birth defects like congenital heart disease, hypospadias and esophageal atresia/stenosis [67-69].

**Strength and limitations of the study:** the strength of this meta-analysis is the first of its kind in sub-Saharan African country and it lies in the quest of existing and unpublished research and the use of several thoughts to digest the study. However, all articles found to have been cross-sectional in nature in this systematic review and meta-analysis. As a consequence, it is not possible to establish temporal relations between factors and outcome variables. Most of the research included in this review had a small sample size that could influence the final estimate. Furthermore, since this meta-analysis included accessible research recorded from a small region in sub-Saharan African countries, the various countries in the study area may be under-represented.

## Conclusion

The present review has revealed a relatively high prevalence of birth defects among newborn infants in sub-Saharan African countries. The most common types of birth defect were musculoskeletal defects, followed by neural tube defects, cardiovascular system defects, gastrointestinal defects and oro-facial clefts (OFCs). Lack of folic acid supplementation, presence of chronic disease and intake of drugs during pregnancy were significantly associated with birth defects. Therefore, based on the findings, it is recommended that efforts should be made to ensure that women use folic acid during the periconceptional period and during pregnancy. In addition, drugs should cautiously be prescribed to pregnant women, and chronic diseases of women must properly be managed so as to minimize the occurrence of birth defects. Furthermore, given the high prevalence of birth defects shown in this review, it is recommended that Fetal Medicine Unit maybe functional in the countries.

### What is known about this topic

- Birth defects are a major public health challenge worldwide, especially in developing countries including Ethiopia;
- Different and few studies have been conducted in the study area;
- The problem is still higher and abundant discrepancy and inconstancy among sub-Saharan African countries.

### What this study adds

- The prevalence of birth defects in sub-Saharan African countries among newborn infants is 20.4 per 1000;
- The most common types of birth defect are musculoskeletal systems defects;
- Maternal history of medication during pregnancy, lack of maternal folic acid supplementation and presence of chronic disease during pregnancy are the factors associated with birth defects.

## Competing interests

The authors declare no competing interests.
